# Locating the ulnar nerve during elbow arthroscopy using palpation is only accurate proximal to the medial epicondyle

**DOI:** 10.1007/s00167-018-5108-y

**Published:** 2018-08-23

**Authors:** Nick F. J. Hilgersom, Davide Cucchi, Francesco Luceri, Michel P. J. van den Bekerom, Luke S. Oh, Paolo Arrigoni, Denise Eygendaal, ESSKA Elbow and Wrist Study Collaborative, Raul Barco, Andrea Celli, Enrico Guerra, Nicolas Holzer, Andreas Lenich, Simone Nicoletti, Luigi Pederzini, Hakan Turan Çift, Kilian Wegmann, Oskar Zupanc, Denise Eygendaal, Paolo Arrigoni

**Affiliations:** 1 Department of Orthopaedic Surgery, Academic Medical Center, University of Amsterdam, Amsterdam Movement Sciences, 1105 AZ Amsterdam, The Netherlands; 2 Department of Orthopaedic Surgery, Sports Medicine Service, Massachusetts General Hospital, Boston, MA USA; 3 Department of Orthopaedics and Trauma Surgery, University of Bonn, Sigmund-Freud Str. 125, 53127 Bonn, Germany; 4 Laboratory of Applied Biomechanics, Department of Biomedical Sciences for Health, Università degli Studi di Milano, Via Mangiagalli 31, 20133 Milan, Italy; 5 Clinica Ortopedica CTO, ASST Centro Specialistico Ortopedico Traumatologico Gaetano Pini-CTO, Piazza Cardinal Ferrari 1, 20122 Milan, Italy; 6 Department of Orthopaedic Surgery, Onze Lieve Vrouwe Gasthuis, 1091 AC Amsterdam, the Netherlands; 7 Department of Orthopaedic Surgery, Amphia Hospital, 4819 EV Breda, the Netherlands

**Keywords:** Elbow, Ulnar nerve, Arthroscopy, Physical examination, Nerve injury, Complication, Prevention

## Abstract

**Purpose:**

Knowledge of ulnar nerve position is of utmost importance to avoid iatrogenic injury in elbow arthroscopy. The aim of this study was to determine how accurate surgeons are in locating the ulnar nerve after fluid extravasation has already occurred, and basing their localization solely on palpation of anatomical landmarks.

**Methods:**

Seven cadaveric elbows were used and seven experienced surgeons in elbow arthroscopy participated. An arthroscopic setting was simulated and fluids were pumped into the joint from the posterior compartment for 15 min. For each cadaveric elbow, one surgeon was asked to locate the ulnar nerve solely by palpation of the anatomical landmarks, and subsequently pin the ulnar nerve at two positions: within 5 cm proximal and another within 5 cm distal of a line connecting the medial epicondyle and the tip of the olecranon. Subsequently, the elbows were dissected using a standard medial elbow approach and the distances between the pins and ulnar nerve were measured.

**Results:**

The median distance between the ulnar nerve and the proximal pins was 0 mm (range 0–0 mm), and between the ulnar nerve and the distal pins was 2 mm (range 0–10 mm), showing a statistically significant difference (*p* = 0.009). All seven proximally placed pins (100%) transfixed the ulnar nerve versus two out of seven distally placed pins (29%) (*p* = 0.021).

**Conclusions:**

In a setting simulating an already initiated arthroscopic procedure, the sole palpation of the anatomical landmarks allows experienced elbow surgeons to accurately locate the ulnar nerve only in its course proximal to the medial epicondyle (7/7, 100%), whereas a significantly reduced accuracy is documented when the same surgeons attempt to locate the nerve distal to the medial epicondyle (2/7, 29%; *p* = 0.021). Current findings support the establishment of a proximal anteromedial portal over a distal anteromedial portal to access the anterior compartment after tissue extravasation has occurred with regard to ulnar nerve safety.

## Introduction

Elbow arthroscopy is a safe and established surgical technique, but nerve injuries may occur, also in the hands of experienced surgeons [2, 5, 13, 15, 20]. In the past two decades, incidences of nerve injury after elbow arthroscopy have been reported between 1.7% and 5.4% [7, 13, 15, 18, 20]. Nerves are easily injured during elbow arthroscopy due to their close relation with the elbow joint capsule, which forms only a thin barrier between the arthroscopic working space and the nerves, and arthroscopic portals that pass the nerve with only millimetres of distance [1, 19].

Since portal placement is a possible cause of ulnar nerve injury, the knowledge of the distance between the ulnar nerve and the arthroscopy portals is of critical importance for safe surgery: when the nerve follows its common course, the distance to the proximal anteromedial portal (AMP) ranges between 12 and 21 mm, with the smallest reported distance being 3–4 mm [1, 28, 30], whereas that to the distal AMP ranges between18 and 25 mm, with the smallest reported distance being 15mm [1, 16, 30].

Therefore, to prevent ulnar nerve injury, the course of the ulnar nerve should always be known prior to AMP placement [11]. Some authors advocate that the ulnar nerve should always be isolated before portal placement, whereas others rely on anatomical landmarks to identify a ‘safe working zone’ [22, 25].

This “safe working zone” is commonly determined at the beginning of the arthroscopic procedure; however, as the duration of the arthroscopic procedure increases, fluid extravasation and tissue swelling may hide the anatomical landmarks used to locate the nerve or move their projection previously marked on the skin, hence confounding the surgeon and posing additional risk of nerve injury; this may happen, for example, if anterior portals have to be placed after a long procedure in the posterior compartment or if an additional anteromedial portal has to be created to assist an anterior procedure (e.g. arthroscopically assisted radial head or coronoid fracture fixation) several minutes after its beginning.

To the authors’ knowledge, the accuracy of locating the ulnar nerve based on palpation has never been quantified before, in particular not after tissue extravasation has occurred. In recent years, Sahajpal et al. [25] and Park et al. [22] proposed the use of an algorithm based on palpation of the ulnar nerve to decide what portal placement technique to use to avoid ulnar nerve injury and successfully used this algorithm in clinical practice; however, this algorithm is applied prior to tissue extravasation.

The aim of this study was to determine how accurate surgeons are in locating the ulnar nerve after tissue extravasation has already occurred, moreover, basing their localization solely on palpation of anatomical landmarks. The study hypothesis is that the ulnar nerve can be located precisely by more than 75% of the surgeons based on palpation of anatomical landmarks.

## Materials and methods

**Fig. 1:**
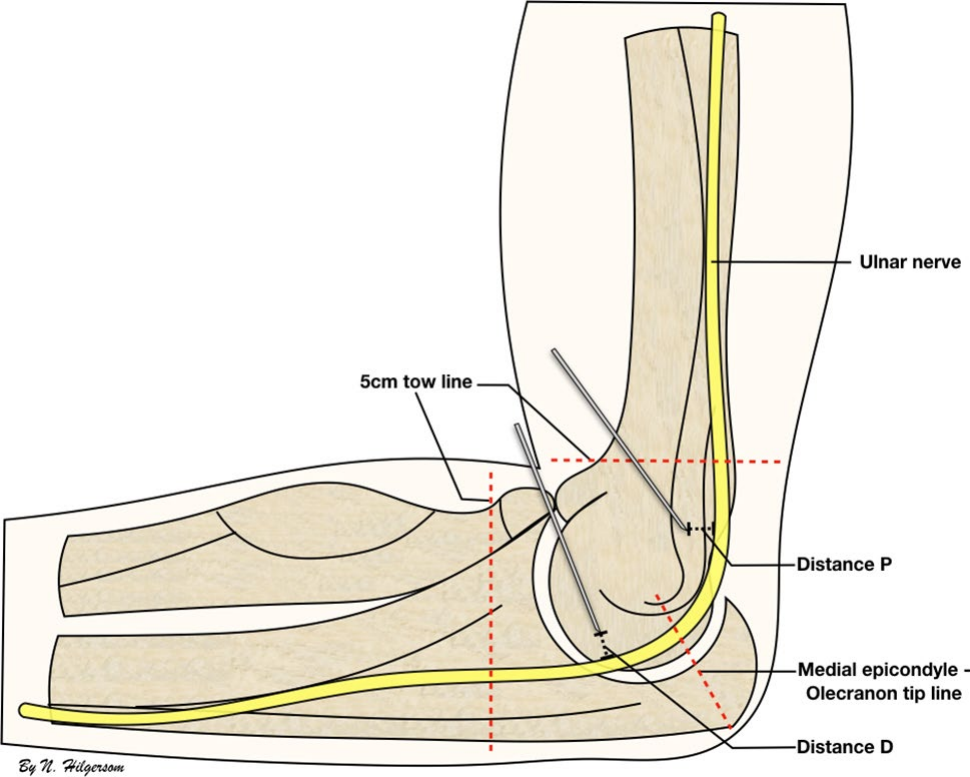
Schematic representation of the setup and pinning of the ulnar nerve. Schematic representation of the medial side of the elbow showing the line between the medial epicondyle and the olecranon tip, the tow lines, distance P [distance between the proximal pin and ulnar nerve, (mm)] and distance D [distance between the distal pin and ulnar nerve (mm)]

Seven fresh-frozen upper limb cadaveric specimens were prepared to mimic an arthroscopic setting. Care was taken in evaluating the specimens for any visible signs of previous trauma, gross instability, deformity or signs of previous surgery. None of the elbow specimens had a (sub)luxating or transposed ulnar nerve. Prior to the beginning of the study, three lines were marked on the skin: the first line connecting the olecranon’s tip with the medial epicondyle; the second and the third parallel to the first one, 5 cm proximal and 5 cm distal. These lines were used to determine the regions in which the ulnar nerve would have to be located (Fig. 1).

Arthroscopy was performed with the elbow positioned at 90° of flexion, with the hand and forearm hanging free with only gravity force. Standard posterolateral and midlateral portals were established. To reproduce a realistic arthroscopic setting with tissue swelling, fluids were pumped in the joint for 15 min, without moving the instruments to the anterior compartment and without creating anterior portals. Subsequently, seven fellowship-trained elbow surgeons were asked to locate the ulnar nerve only by palpating anatomical landmarks (e.g. medial epicondyle, medial intermuscular septum). For each cadaveric specimen, one surgeon was asked to try to transfix the ulnar nerve with two pins, in two different locations: first within 5 cm proximal to the medial epicondyle, and then within 5 cm distal to it, as previously marked with parallel lines on the skin. After each specimen was transfixed with two pins, all specimens were dissected using a standard medial approach to expose the cubital tunnel. The ulnar nerve was then identified without removal of the pins and two distances were measured with a graduated calliper allowing accurate measurements with 1 mm accuracy: between the proximal pin and the ulnar nerve (distance P), and between the distal pin and the ulnar nerve (distance D). An external observer, not included among the examiners and not involved in the statistical evaluation of the collected data, measured each distance in triplicate. The averages of all three measurements per distance were used for statistical analysis. In addition, the trans-epicondylar distance was measured in each cadaver specimen to provide an indication about the elbow size.

Institutional approval of the study protocol was obtained by the Nicola’s Foundation & ICLO Research Center (ID10607).

### Statistical analysis

Data analysis was performed using Stata, version 14.0 (StataCorp LP, College Station, TX, USA). The pin-to-nerve distances are reported as mean and standard deviation (SD) or median and range, depending on the normality of the data. In bivariate analysis, a Student’s *t* test or Mann–Whitney *U* test, depending on normality of the data, was used to compare distance P and distance D. In addition, the pin-to-nerve distances were dichotomized into hits (distance of 0 mm) and misses (distance > 0 mm). A Fisher’s exact test was used to compare the proportions of hits proximal and distal to the line between the medial epicondyle and olecranon tip. A *p* value of < 0.05 was considered statistically significant.

Since the number of anatomical studies dealing with the mutual relation between anteromedial portals and ulnar nerve is limited, the sample size for this study was based on previous publications regarding anatomical dissections of the more frequently studied posterior interosseous nerve: this was successfully investigated also on relatively small groups of specimens in open, arthroscopic and imaging-controlled studies [3, 9, 17, 29]. Based on these experiences, a minimum of five specimens was considered as suitable to conduct this study. The number of experienced elbow arthroscopy surgeons available at the moment of study conduction limited the sample size to a final number of seven.

## Results

Seven fresh-frozen elbow specimens (females 86%, left elbow 43%, median inter-epicondylar distance: 6 mm) were evaluated by seven surgeons. The demographics of the surgeons are shown in Table 1.

**Table 1:** Demographics of the surgeons

Expert	Gender	Age (years)	Experience (years)	Number of arthroscopies (past 12 months)
1	Male	41	10	80
2	Male	48	14	24
3	Female	48	17	40
4	Male	47	15	100
5	Male	45	9	50
6	Male	61	27	70
7	Male	39	5	45
	Median	47	14	50
	Range	39–61	5–27	24–100

All seven pins (100%) placed proximally to the medial epicondyle transfixed the ulnar nerve (distance P = 0 mm). Only two out of seven (29%) pins placed distally to the medial epicondyle transfixed the ulnar nerve. The median distance D was 2 mm (range 0–10 mm). Bivariate analysis showed a statistically significant difference between both distance P and distance D (*p* = 0.0091), and the number of pins transfixing the ulnar nerve proximal and distal to the line between the medial epicondyle and olecranon top (*p* = 0.021) (Table 2).

**Table 2:** The pin-to-nerve distances measured for each surgeon

Surgeon data			Elbow specimen data (*n* = 8)		
Surgeon	Distance P (mm)	Distance D (mm)	Laterality	Sex	Trans-epicondylar distance (cm)
1	0	0	Right	Male	6
2	0	10	Left	Female	5.7
3	0	0	Right	Female	6.6
4	0	2	Left	Female	8.2
5	0	1	Right	Female	5.8
6	0	5	Left	Female	4.9
7	0	2	Right	Female	6.4
Median	0	2			
Range	0–0	0–10			
Mann–Whitney *U* test	*p* = 0.0091*				

*Distance P = pin-to-nerve distance proximal to the medial epicondyle

*Distance D = pin-to-nerve distance distal to the medial epicondyle

## Discussion

The main finding of this study is that in a setting simulating an already initiated arthroscopic procedure, the sole palpation of the anatomical landmarks allows experienced elbow surgeons to accurately locate the ulnar nerve only in its course proximal to the medial epicondyle (7/7, 100%), whereas a significantly reduced accuracy in the localization of the nerve is documented when the same surgeons attempt to locate the nerve distal to the medial epicondyle (2/7, 29%; *p* = 0.021). Therefore, our hypothesis that more than 75% of surgeons can accurately locate the ulnar nerve by palpation of anatomical landmarks after fluid extravasation is confirmed for the proximal localization and is rejected for the distal one.

**Fig. 2:**
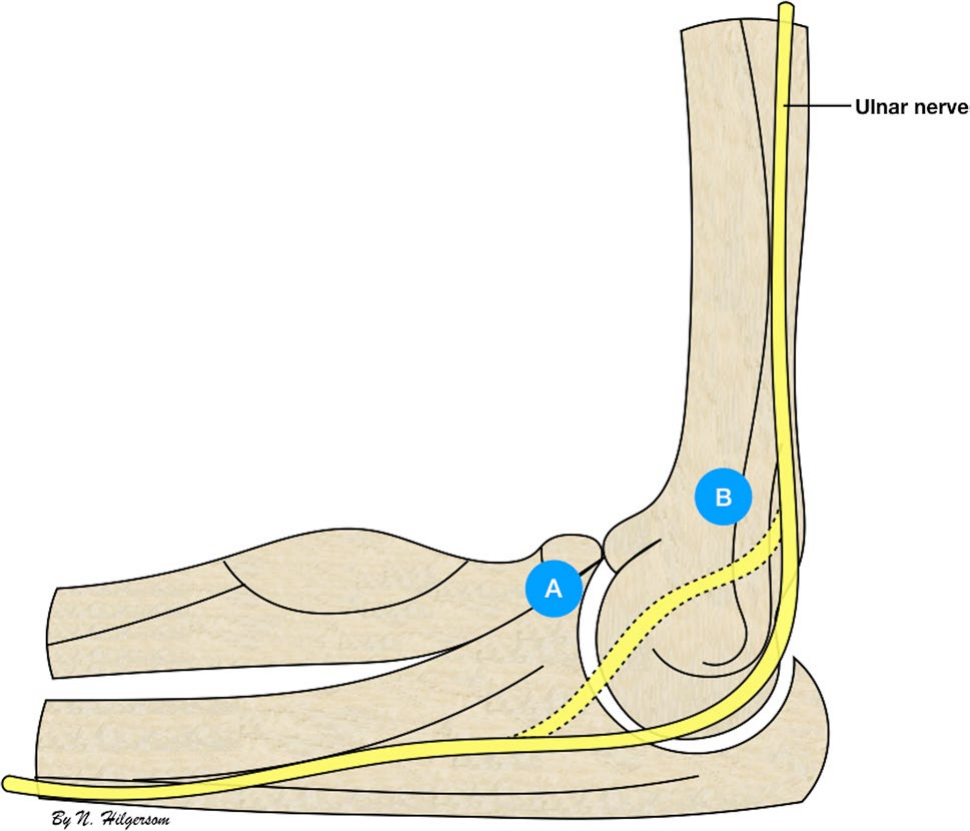
The ulnar nerve and anteromedial portals. Schematic representation of the medial side of the elbow showing the anatomic course of the ulnar nerve (continuous line) and a luxating or transposed ulnar nerve (dashed line) in relation to the distal anteromedial portal (A), 2 cm distal and 2 cm anterior to the medial epicondyle, and proximal anteromedial portal (B), 2 cm proximal and just anterior to the medial intermuscular septum

Nerves at risk during elbow arthroscopy are the radial, median, ulnar, lateral antebrachial cutaneous and medial antebrachial cutaneous nerve. Approximately, 38–42% of nerve injuries associated with elbow arthroscopy involve the ulnar nerve [5, 15]. The ulnar nerve is frequently injured by direct trauma, which can occur by posteromedial or AMP placement (Fig. 2), or using motorized instruments inside the joint in close proximity to the medial capsule [6–8, 10, 12]. The latter may lead to accidental entanglement or suction injury of the ulnar nerve, as the medial capsule forms only a thin barrier between the arthroscopic working space and the nerve. The posteromedial portal is generally not used, as it directly overlies the ulnar nerve [21, 28].

**Fig. 3:**
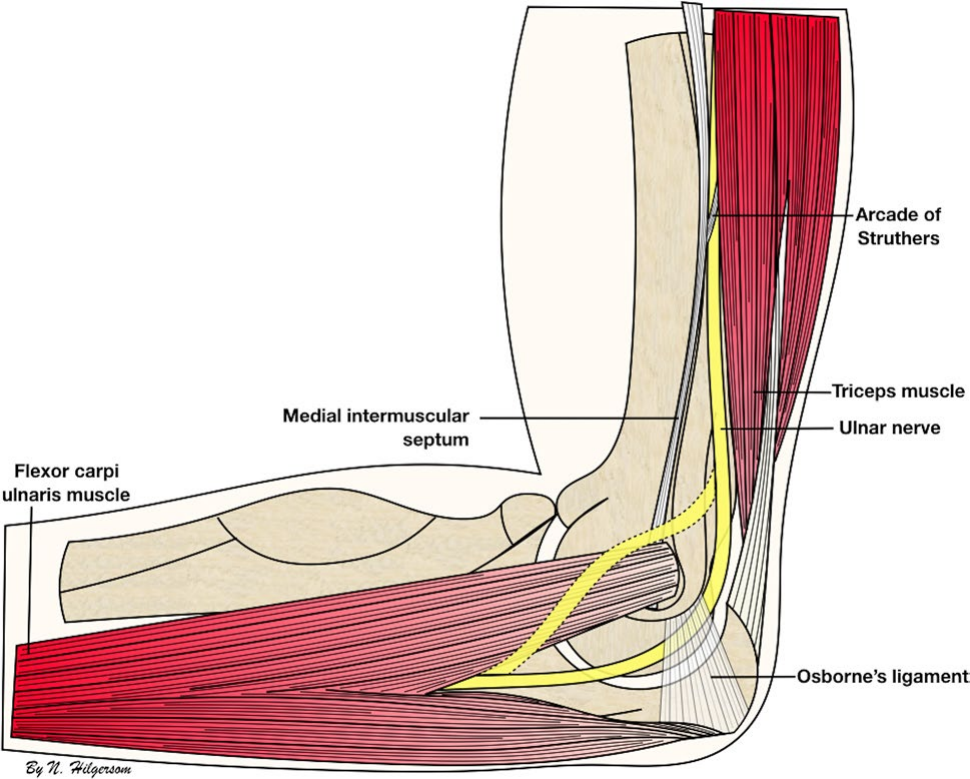
Ulnar nerve anatomy. Schematic representation of the medial side of the elbow showing the anatomic course of the ulnar nerve (continuous line) and the course of a luxating or transposed ulnar nerve (dashed line), including relevant anatomic features along its course

Anatomical studies describe that the ulnar nerve, after traversing through the arcade of Struthers, runs posteriorly to the medial intermuscular septum in the upper arm and then courses posteriorly to the medial epicondyle to enter the cubital tunnel (Fig. 3) [14, 24, 27]. However, in the presence of a luxating or subluxating ulnar nerve, a previously transposed ulnar nerve or in the rare cases in which the ulnar nerve runs anterior to the medial epicondyle, the ulnar nerve may not be where expected and the nerve-to-portal distances may be smaller [8, 26]. These instances pose extra risk for iatrogenic ulnar nerve injury during anteromedial portal placement (Figs. 2, 3).

Based on the high accuracy found for proximal localization of the ulnar nerve, experienced elbow surgeons appeared capable of determining a safe proximal AMP site using palpation of the ulnar nerve only, also after beginning of the arthroscopic procedure and joint irrigation. The findings of our study support the algorithm proposed by Park et al. [22], originally designed to guide safe proximal AMP placement in elbows with a transposed ulnar nerve. In this algorithm, patients are subdivided based on the certainty with which the ulnar nerve can be located (certain versus uncertain). In case the ulnar nerve is palpable with certainty, the portal is placed in a standard antegrade fashion, approximately 1 cm away from the ulnar nerve, while holding the ulnar nerve posteriorly or anteriorly if mobile. However, in case the location of the ulnar nerve is doubtful, following this algorithm, a 1–3 cm skin incision with blunt dissection onto the capsule is recommended before portal placement, to prevent iatrogenic injury to the ulnar nerve. Using this algorithm, under the assumption that all surgeons in our study were certain about the proximal location of the ulnar nerve, none of the surgeons would have injured the ulnar nerve while placing a proximal AMP after palpation of the ulnar nerve.

Distal localization of the ulnar nerve was significantly less accurate among the experienced elbow surgeons in this study, with a median pin-to-nerve distance of 2 mm (range 0–10 mm). This finding supports current clinical practice, in which, ever since the introduction of the proximal AMP by Poehling et al. [23], the distal anteromedial portal was abandoned as a starting portal. The distal AMP is currently only used as a secondary portal, established using an inside-out technique to avoid injury to the median nerve. The significant lower accuracy in locating the ulnar nerve in this study further supports the use of an inside-out technique for establishment of a distal AMP, to protect the median as well as the ulnar never from iatrogenic damage.

The fact that the measurements in this study were performed after 15 min of joint irrigation further supports this indication, since fluid extravasation and soft tissue swelling appeared to critically compromise the palpability of the ulnar nerve in its distal portion, leading the majority of the involved surgeons to miss the ulnar nerve. Therefore, if the creation of an accessory anteromedial portal is necessary after beginning of the arthroscopic procedure, it is recommended to isolate and identify the ulnar nerve.

The difference in accuracy of locating the ulnar nerve with palpation proximally and distally may be explained by numerous factors: the anatomic trajectory of the ulnar nerve, the presence of the medial intermuscular septum and the tissue swelling. The ulnar nerve runs relatively superficial proximal to the medial epicondyle, as it is not covered by any fascia or ligament after it has passed Struther’s ligament, while distal to the medial epicondyle, the ulnar nerve traverses the cubital tunnel underneath Osborne’s ligament and Osborne’s fascia, and continues beneath the aponeurosis of the humeral and ulnar heads of the flexor carpi ulnaris muscle [14, 24, 27], possibly making it harder to palpate. In addition, the well-palpable medial intermuscular septum can be used as a reliable anatomic landmark proximal of the medial epicondyle, whereas a similar anatomic reference distal to the medial epicondyle is lacking. Finally, in a setting simulating a lateral decubitus position with the elbow positioned at 90° of flexion and the forearm hanging free with only gravity force, the extravasation of fluid into the periarticular soft tissues may be more prominent in the distal part.

Several limitations should be considered for this study. First, we examined a relatively small group of experienced elbow surgeons. Therefore, the accuracy of ulnar nerve identification found in this study may not apply to less experienced surgeons. Claessen et al. [4] found a significantly higher complication rate with portal placement by novice surgeons compared to reported complication rates by experienced elbow surgeons [7, 18]. Secondly, the number of surgeons participating in this study and the number of cadaveric specimens were small.

To the best of our knowledge, this is the first study examining the accuracy of surgeons in ulnar nerve localization by palpation in a simulated arthroscopic setting after several minutes of joint irrigation. Our findings confirm that experienced elbow surgeons are capable of accurate localization of the ulnar nerve proximal to the medial epicondyle using solely palpation after tissue extravasation has occurred, allowing safe proximal AMP placement based solely on palpation of the ulnar nerve in the swollen elbow. Their localization of the ulnar nerve distal to the medial epicondyle was significantly less accurate, supporting the current practice of using an inside-out technique for distal AMP placement. The relevance of the current study for daily clinical practise is that it shows the preferential use of a proximal anteromedial portal over a distal anteromedial portal to access the anterior compartment after tissue extravasation had occurred with regard to ulnar nerve safety. Nonetheless, it remains paramount for a surgeon to remain self-critical, and if doubtful about the location of the ulnar nerve to use a skin incision and blunt dissection onto the capsule, to avoid the risk of iatrogenic nerve injury because of portal placement.

## Conclusions

In a setting simulating an already initiated arthroscopic procedure, the sole palpation of the anatomical landmarks allows experienced elbow surgeons to accurately locate the ulnar nerve only in its course proximal to the medial epicondyle (7/7, 100%), whereas a significantly reduced accuracy in the localization of the nerve is documented when the same surgeons attempt to locate the nerve distal to the medial epicondyle (2/7, 29%; *p* = 0.021). Relevance for daily clinical practice is the support for use of a proximal anteromedial portal over a distal anteromedial portal to gain access to the anterior elbow compartment after tissue extravasation has occurred with regard to ulnar nerve safety.

## Abbreviations

AMP: Anteromedial portal
Distance P: Pin-to-nerve distance proximal to the medial epicondyle
Distance D: Pin-to-nerve distance distal to the medial epicondyle
SD: Standard deviation
